# A Clinical Decision Support System for the Diagnosis, Fracture Risks and Treatment of Osteoporosis

**DOI:** 10.1155/2015/189769

**Published:** 2015-03-01

**Authors:** Bjarni V. Halldorsson, Aron Hjalti Bjornsson, Haukur Tyr Gudmundsson, Elvar Orn Birgisson, Bjorn Runar Ludviksson, Bjorn Gudbjornsson

**Affiliations:** ^1^Institute of Biomedical and Neural Engineering, School of Science and Engineering, Reykjavik University, 101 Reykjavik, Iceland; ^2^Centre for Rheumatology Research, University Hospital, 101 Reykjavik, Iceland; ^3^Faculty of Medicine, Debrecen University, Debrecen 4032, Hungary; ^4^Faculty of Medicine, University of Iceland, 101 Reykjavik, Iceland; ^5^Osteoporosis Clinic, Akureyri Hospital, 600 Akureyri, Iceland; ^6^Department of Immunology, University Hospital, 101 Reykjavik, Iceland

## Abstract

Expanding medical knowledge increases the potential risk of medical errors in clinical practice. We present, OPAD, a clinical decision support system in the field of the medical care of osteoporosis. We utilize clinical information from international guidelines and experts in the field of osteoporosis. Physicians are provided with user interface to insert standard patient data, from which OPAD provides instant diagnostic comments, 10-year risk of fragility fracture, treatment options for the given case, and when to offer a follow-up DXA-evaluation. Thus, the medical decision making is standardized according to the best expert knowledge at any given time. OPAD was evaluated in a set of 308 randomly selected individuals. OPAD's ten-year fracture risk computation is nearly identical to FRAX (*r* = 0.988). In 58% of cases OPAD recommended DXA evaluation at the present time. Following a DXA measurement in all individuals, 71% of those that were recommended to have DXA at the present time received recommendation for further investigation or specific treatment by the OPAD. In only 5.9% of individuals in which DXA was not recommended, the result of the BMD measurement changed the recommendations given by OPAD.

## 1. Introduction 

According to the International Osteoporosis Foundation (IOF), one in three women and one in five men will experience an osteoporotic fracture later in their life; thus, globally an osteoporotic fracture is estimated to occur every third second [1–3]. In that context, nine million North Americans have osteoporosis and it is estimated that 43 million have low bone mass measured by DXA (http://nof.org/news/1648), a precursor of osteoporosis, known as osteopenia. The National Osteoporosis Foundation (NOF) in the USA has estimated that, in USA alone, osteoporosis related fractures number two million every year resulting in an annual cost of $19 billion [4]. Due to the increasing numbers of elderly people, the number of fractures will increase to three million in 2025, resulting in a $25.3 billion annual cost in the USA [5]. Correctly prescribed bone protective treatment may reduce the fracture risk by 30–50% in only three years [6]. It is therefore critical to identify individuals at risk of a fragility fracture and offer them the treatment protocols to decrease the number of fractures in coming years.

The diagnosis of osteoporosis has traditionally relied on bone mineral density (BMD) measurement [7–9]. However, a number of other factors affect a physicians' decision to treat osteoporosis, including family history, lifestyle, and various medical factors [10]. The incorporation of all of these factors into a treatment recommendation and a decision to perform a BMD scan can be a formidable task that requires considerable expert knowledge and experience. Here we describe the development of a clinical decision support system that automates the dissemination of this knowledge.

Currently available risk calculator systems for osteoporosis, for example, FRAX [11], Garvan [12], and Qfracture [13], provide the user with ten-year probability of fragility fracture related to osteoporosis. While this information may be useful for a number of users, fracture risk is known to be difficult for the layperson to interpret [14] and even for nonexpert health professionals. Furthermore, it is not immediately evident how this information is to be interpreted, for example, for treatment decision in daily clinical practice. Clinical decision support systems (CDSS) have been suggested as a means of disseminating knowledge of best practice to physicians [15], electronic medical reminders have been shown to improve osteoporosis management after fractures [16], and Kastner and Straus [17] have concluded that “multi-component tools that are targeted to physicians and patients may be effective for supporting clinical decision making in osteoporosis disease management.”

We have developed a clinical decision support system that gives the 10-year fracture probability due to osteoporosis for the individual patient, lifestyle recommendations, and recommendations as to whether and when a BMD scan is recommended. Furthermore, the system will identify patients at risk of fractures and would benefit from specific preventive medical treatment. Our osteoporosis adviser (OPAD) is designed for those with medical knowledge such as general physicians and clinical nurse specialists.

In this paper, we evaluate the reliability of the OPAD system by comparing its 10-year fracture probability with the probability given by FRAX and by assessing the quality of its BMD scanning recommendations, that is, whether those that were recommended to have a BMD measurement benefited from the measurement.

## 2. Materials and Methods

### 2.1. The Osteoporosis Advisor (OPAD)

We have designed an expert system to assist in the diagnosis and treatment of osteoporosis. The software takes as input a set of clinically relevant parameters from which the 10-year fracture risk is computed based on published country specific data [8, 11, 18]. The output of the program is as follows: the 10-year fracture risk of an individual, lifestyle and treatment recommendations, and lastly a suggestion for the time when a follow-up BMD scan should take place. The clinically relevant parameters and the computed risk for fracture are used as input into an expert system which gives specific recommendations for each case with respect to lifestyle changes and treatment options.

The software further outputs immediately relevant information to the user: a risk group for the individual (low, medium, or high risk for fracture compared to age-matched controls [18, 19]) and a diagnosis of osteopenia, osteoporosis, or manifested osteoporosis (osteoporosis with fracture), as well as for glucocorticosteroid induced osteoporosis. Diagnosis of osteoporosis is made according to the WHO definitions [20], where osteoporosis is diagnosed when BMD results in a *T* value of −2.5 or lower; that is, the BMD is ≥2.5 SD below the mean of young individuals of the same sex and reach, and osteopenia is diagnosed when the *T* value is between −1 and −2.5.

### 2.2. Design of Expert System

The design of the system uses a knowledge mapping approach. Expert physicians were queried to determine the clinically relevant parameters for the recommendation of osteoporosis treatment and recommendations for BMD measurements. A group of different specialists who were all interested in osteoporosis (rheumatologist, endocrinologist, general practitioner, and geriatrician) participated in the process. The Intellix Advisor [21, 22] was used for knowledge capture in the model.

The Intellix Advisor allows for active acquisition of knowledge. A set of instances is input into the software and from these input examples the software can be told either to construct a neural network based model based on the examples or to ask for more examples that consist of patients not considered by the model. In our approach patients were added to the model until a diagnosis or recommendations could be made for every possible tested patient. The model is implemented as a lookup table and for every new patient diagnosed a patient with the same characteristics is found in our database.

The diagnosis for a patient includes four distinct pieces of information: 10-year fracture risk, lifestyle recommendations, treatment recommendations, and recommendation of the time for the next BMD measurement or follow-up evaluation of the individual patient. Initially a set of clinically relevant parameters was determined (see Table 1).

### 2.3. Knowledge Capture of Treatment Recommendations

The OPAD system follows the frame of international guidelines, for example, the Scottish Intercollegiate Guidelines Network (SIGN) on osteoporosis (http://sign.ac.uk/pdf/sign71.pdf), and the system also takes into account regional differences, that is, local or national guidelines. However, the system predominantly relies on knowledge capture process of the expert panels, as guidelines never cover all cases. In the end a total of fifteen different treatment recommendations were initially identified as possible recommended treatment options for osteoporosis. The recommended treatments ranged from no treatment to specific recommendations for which drug class was the most appropriate, either as a preventive measure or as a treatment for manifest osteoporosis.

Table 2 lists the clinically relevant patient attributes determined by our expert physicians. Different medical specialists with expertise in osteoporosis reviewed a list of cases that had recently visited the osteoporosis clinic at the University Hospital in Reykjavik (LSH), Iceland. For each patient the physicians reviewed their clinical decision and were then asked to determine which of the clinically relevant parameters influenced his decision. The clinically relevant information and the diagnoses were entered into the Intellix Advisor, leaving those clinical relevant parameters that were not relevant for the decision as “do not care.” After considering the set of real patient cases and their detailed experts' reviews, the Intellix Advisor software constructed a set of virtual patients having clinical characteristics not observed among the list of cases already considered. This process was continued until the space of all possible patients was covered; a decision could be reached for all possible patients that could enter the clinic, independent of clinical characteristics. As a result a total of 80 relevant rules were constructed in the final outcome of the system.

### 2.4. Time until Next DXA Measurement

The recommended time until the next DXA measurement was also determined by using a knowledge mapping process. A list of clinically relevant pieces of information was determined which can be seen in Table 3.

Seven different recommendations were made for the time for the next BMD measurement, listed in Table 4. The construction of the clinical decision model then followed the same protocol as described above for the treatment recommendations. In the end a total of 87 rules were determined to be clinically relevant.

### 2.5. Capture of Disease Risk Models

The computed 10-year risk of fracture was based on the World Health Organization (WHO) fracture risk assessment recommendations [19, 20]. Thus, the fracture risk predications delivered by our system are comparable to the results given by FRAX [18]. The output of the FRAX in collaboration with NOGG recommendation guidelines also includes the classification of individuals into high, medium, and low risk individuals [23]. The FRAX recommendation guidelines are given as a set of text tables, with the risk of fracture and confidence interval for the risk of fracture given as a function of age and the number of risk components an individual has. As the tables only give fracture risk probabilities for selected age groups, an interpolation is done to compute the fracture risk probabilities for other age groups.

### 2.6. Model Testing

In order to validate that the treatment recommendations presented to the end user agreed with the treatment recommendations originally determined for each patient a quality control module was developed. Thus, built on top of our clinical database a set of 300 virtual quality control patients was created which were used to automatically verify the correctness of the system recommendations for real life clinical information given for each case. Experiments verified that the results of these patients agreed in both the model created and the interface to the end user.

### 2.7. Test in Real Life

As the WHO recommendation guidelines are not given with a closed form formula we compared the fracture risk computation given by our OPAD system with the fracture risk computation given by FRAX. We selected consecutive 308 individuals from the out-patient osteoporosis clinic at LSH, who visited the clinic from the 1st of January 2012 onwards. We compared the ten-year fracture risk computed using the OPAD and the ten-year fracture risk computed using recommendations given by our model with those given by FRAX. Linear regression was run to compare the results between the two systems.

We also reevaluated the same group of 308 individuals with our OPAD system with respect to the need of DXA at the present time or later; that is, the risk evaluation was done without the DXA results (*T* value). We then analyzed whether the DXA result, that is, in those cases where OPAD did not recommend DXA at the given time, influenced the automatic treatment and the follow-up recommendations given by the OPAD compared to the recommendations by the experts, who had access to all the clinical data.

### 2.8. Ethics Statement

The Data Protection Authority (S5680) and the National Bioethics Committee of Iceland approved the study protocol (VSNb2010050008). The original patients' data were hosted by the University Hospital, Reykjavik, Iceland. The data were anonymized before being provided to the researchers.

## 3. Results

### 3.1. Correlation between Risk Evaluation by OPAD and FRAX

**Figure 1:**
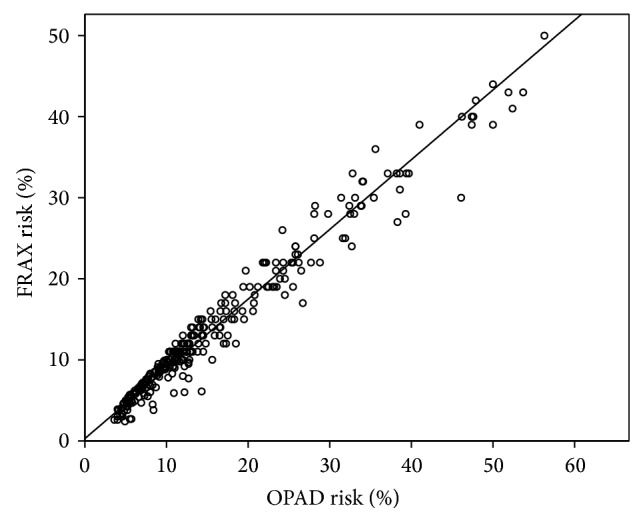
Ten-year risk for major osteoporotic fracture computed using the osteoporosis advisor (OPAD; *x*-axis), compared to the fracture risk computed using FRAX (*y*-axis).

Of the 308 cases, 39 were males and 269 were females, with a mean age of 61 years (15–89).  Figure 1 shows the 10-year fracture risk when DXA is included presented by the two different programs, that is, OPAD and FRAX. We obtained a correlation of *r* = 0.988 with a mean paired difference of 0.7678% (SD = 1.9946%) when comparing individual patient prior to DXA evaluation and *r* = 0.977, mean difference 1.8285% (SD = 2.8%) when DXA result were included in the risk evaluation or a near perfect correlation between these two risk calculators.

### 3.2. OPAD and Next DXA

These 308 patients were reevaluated by the OPAD with respect to the need for a DXA at the present time or later, that is, before they underwent their DXA evaluation.

In 178 cases (58%), out of these 308 cases, the OPAD system recommended DXA evaluation at the present time. Following DXA measurement in these 178 cases, 91 (51%) of those received OPAD recommendation on specific treatment options, where 5 patients (3%) were recommended to continue with their treatment. Additional 31 patients (17%) received recommendation on consulting specialist in osteoporosis. Meanwhile, only 51 of these 178 cases (29%) received general prevention measurement recommendations. Thus, the DXA investigation performed according to the recommendation of the OPAD system seems to influence the clinical decision-making process.

Out of the 308 original cases, 102 cases (33%) came for their DXA even though the OPAD system would have recommended that they should have their DXA at the age of 65; that is, every third patient who came in for a measurement did not need the DXA evaluation at the present time according to the OPAD system.

### 3.3. Influence of DXA on OPAD Recommendations

In only six of these 102 cases (5.9%), did the OPAD system change its recommendation following the BMD measurement? In four cases the OPAD recommended specific bone protective treatment, due to the fact that these four individuals were diagnosed with osteoporosis with a significantly increased risk of fragility fracture, that is, with a 10-year fracture risk in the range from 9.1% to 14.3%. In two additional cases the OPAD changed its recommendation to continuation of already taken measurements.

In a further 22 cases of these 308 cases (7.1%) the OPAD system recommended DXA within 1–3 years depending on various clinical circumstances. Independent of these recommendations all patients received a DXA measurement, like other patients in this study and only one of these individuals received a different recommendation following the DXA evaluation.

## 4. Discussion

Osteoporosis is a disorder affecting the density and infrastructure of the bone mass [20]. In its worst outcome it can lead to the so-called fragility fractures [24], most frequently seen in the vertebral spine, wrist, and the hip. Due to their critical location, such fractures most often result in a debilitating outcome for those affected individuals leading to a significantly negative impact on not only their quality of life but also individual life expectancy [25]. Despite an effective drug treatment being readily available for affected patients, it has been recognized that the majority of individuals at risk and also those already affected with osteoporosis and increased risk of fragility fracture are not correctly diagnosed or go unnoticed and therefore miss potentially lifesaving therapeutic measures [6]. Thus, even patients who have suffered fragility fractures and have been exposed to the healthcare system are not identified and treated with the proper preventive regimen they deserve. In this context, if correctly implemented clinical decision support systems such as the one presented here (OPAD) should have the potential to improve both public and healthcare workers awareness of osteoporosis [15]. Such measures not only would improve and streamline the diagnosis process but would also become a valid diagnosis aid to secure the optimal treatment and outcome of our osteoporotic patients.

Bone mineral density (BMD), that is, bone mass, may be measured with several methods, for example, quantitative ultrasound (QUS), peripheral quantitative computer tomography (pQCT), and dual energy X-ray absorptiometry (DXA) which is the golden standard of bone mass measurement. Most professional interest groups, for example, NOF and IOF, have published recommendations on when to use DXA for BMD measurement [26], while others research groups supported by the National Institutes of Health have made an effort to analyze the need of rescreening in postmenopausal women [27]. As osteoporosis is a silent disease until the fracture occurs, BMD measurement is the only method to diagnose osteoporosis prior to the fracture. However, the access to DXA-machines is limited in most countries, and it is therefore important to find those at risk of osteoporosis for DXA-evaluation and those individuals who receive the greatest benefits from the investigation. Thus, improving the cost efficiency by correctly identifying those at risk and those who should be referred for BMD should lead to shorter waiting lists for specialist referrals and improved diagnostic accuracy. Our finding that more than one third of BMD tested patients could be identified a priori to be at no risk of osteoporosis related fractures, thus, did not benefits from the DXA evaluation.

Several risk calculator tools for bone fractures have been presented, but only FRAX has been recommended and supported by the World Health Organization (WHO). FRAX was developed for the calculation of the 10-year fracture risk for hip fracture or a major osteoporotic fracture (clinical spine, forearm, hip, or shoulder fracture) based on certain risk factors, with or without results of DXA measurement of the hip. FRAX offers country specific values for several countries in Europe, North and Latin America, the Middle East, Africa, and Oceania, or a total of 52 country specific datasets [18]. However, FRAX does not give any specific diagnosis or treatment options, and neither have they reported their algorithms used to derive the 10-year fracture risk. Further analysis of our 10-year fracture risk in context to the given recommendation by our OPAD is in progress, before we can make our methodology public in detail.

**Figure 2:**
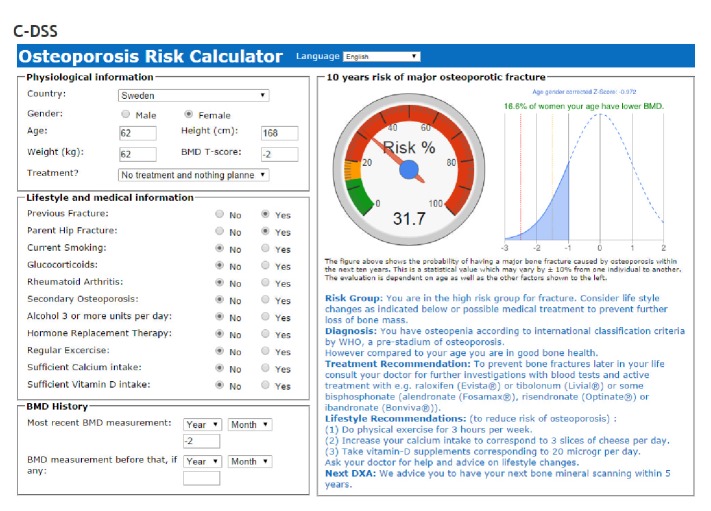
A 62-year-old Swedish female patient with history of fracture whose mother also had a history of hip fracture. She did not have other medical risk factors and she had osteopenia according to a recent DXA evaluation with a *T*-score of −2.0.

The busy clinician may have difficulties in interpreting the risk value figure for each patient in hectic daily clinical practice. With this in mind we have extended the information provided by OPAD, by giving a specific diagnosis, that is, osteoporosis or osteopenia, and specific recommendations on prevention, time of next DXA, and treatment options according to international guidelines and experts knowledge [10]. In addition, our results are presented on an interactive riskometer, which gives a comparison to the background population of the same sex and age, both graphically and by calculation of the *Z* value for the individual patient (please see Figure 2).

Although physicians subscribe to several medical journals, presenting thousands of articles yearly, they have difficulty in keeping up to date in all areas in their daily practice. The more complicated medical world amplifies the risk of diagnosis errors. OPAD allows “best practice” in osteoporosis risk evaluation of fragility fractures and treatment to be captured, distributed, and automated in a simple bedside manner for the busy practicing physician and other health care providers, including nurses working in fracture liaison services, as now highly recommended by IOF [28]. Although the OPAD system presented in this present study reflects Swedish data, it runs in ten different national specific datasets. Improvements in treatment alternatives or changes in clinical guidelines can easily be incorporated into the OPAD system; even country specific guidelines can be internalized. Further studies are needed to analyze whether a clinical approach with the help of digital CDSS tools, such as OPAD, may not only improve individual care, but also become highly cost effective. Such studies need to involve primary care, fracture clinics, and in-hospital fracture liaison services. Furthermore, survey among primary care doctors regarding their use of and evaluation of the OPAD system, including their assessment of how to implement clinical discussion system, such as OPAD, was carried on fracture risk management and treatment in daily clinical primary care praxis.

We conclude that OPAD is accurate in respect to fracture risk probability evaluation and may presumptively be cost effective in fracture liaison services. However, cost-benefit studies are needed in the field of osteoporosis preventive care and CDSS.

## Figures and Tables

**Table 1:** Patient attributes used by the osteoporosis advisor.

Age
Bone mineral density (*T* value)
Ethnicity
Gender
Previous osteoporotic related fracture
Parent hip fracture
Current smoking
Current use of glucocorticosteroids for more than three months
Rheumatoid arthritis
Secondary osteoporosis
Alcohol: 3 or more units per day
Hormone replacement therapy
Regular exercise
Sufficient calcium intake
Sufficient vitamin D intake

**Table 2:** Patient attributes used for the recommendation of osteoporosis treatment.

Attribute	Type
Gender	Male/female
GIOP	Yes/no
Fragility fracture	Yes/no
Fracture risk	High/medium/low
Treatment	Yes/no
Secondary osteoporosis	Yes/no
*T* value	Numerical
Age	Numerical
Menopause status	Before/<3 years/>3 years
Diagnosis	None/osteopenia/osteoporosis/manifest osteoporosis/GIOP

**Table 3:** Attributes used to determine time until next DXA measurement.

Attribute	Type
Gender	Male/female
Menopause	Before, <3 years, >3 years
Risk group	High/medium/low
Treatment	Yes/no
Changes in BMD measured by DXA	No DXA/improving/unknown or losing/fast loosing/neutral
Glucocorticosteroids	Yes/no

**Table 4:** The possible recommendations for the next time for a BMD scan.

At menopause
At the age of 65
Now
In 1-2 years
In 3 years
In 5 years
DXA not recommended
