# Corrigendum to “A Clinical Decision Support System for the Diagnosis, Fracture Risks and Treatment of Osteoporosis”

**DOI:** 10.1155/2017/5472789

**Published:** 2017-06-29

**Authors:** Bjarni V. Halldorsson, Aron Hjalti Bjornsson, Haukur Tyr Gudmundsson, Elvar Orn Birgisson, Bjorn Runar Ludviksson, Bjorn Gudbjornsson

**Affiliations:** ^1^Institute of Biomedical and Neural Engineering, School of Science and Engineering, Reykjavik University, 101 Reykjavik, Iceland; ^2^Centre for Rheumatology Research, University Hospital, 101 Reykjavik, Iceland; ^3^Faculty of Medicine, Debrecen University, Debrecen 4032, Hungary; ^4^Faculty of Medicine, University of Iceland, 101 Reykjavik, Iceland; ^5^Osteoporosis Clinic, Akureyri Hospital, 600 Akureyri, Iceland; ^6^Department of Immunology, University Hospital, 101 Reykjavik, Iceland

In the article titled “A Clinical Decision Support System for the Diagnosis, Fracture Risks and Treatment of Osteoporosis” [1], there was an error regarding the FRAX® tool, which should be clarified as follows.

The article notes: “the computed 10-year risk of fracture was based on the World Health Organization (WHO) fracture risk assessment recommendations [19,20]”; “Several risk calculator tools for bone fractures have been presented, but only FRAX has been recommended and supported by the World Health Organization (WHO).” However, the World Health Organization (WHO) did not develop, test, or endorse the FRAX tool or its recommendations [2]. The metabolic bone disease unit at the University of Sheffield that developed FRAX was a WHO Collaborating Centre from 1991 to 2010, but treatment guidelines must undergo a formal process before they can be endorsed by the WHO.
